# Target Analysis of Volatile Organic Compounds in Exhaled Breath for Lung Cancer Discrimination from Other Pulmonary Diseases and Healthy Persons

**DOI:** 10.3390/metabo10080317

**Published:** 2020-08-03

**Authors:** Michalis Koureas, Paraskevi Kirgou, Grigoris Amoutzias, Christos Hadjichristodoulou, Konstantinos Gourgoulianis, Andreas Tsakalof

**Affiliations:** 1 Department of Hygiene and Epidemiology, University Hospital of Larissa, Faculty of Medicine, University of Thessaly, 22 Papakyriazi Street, 41222 Larissa, Greece; mkoureas@med.uth.gr (M.K.); xhatzi@med.uth.gr (C.H.); 2 Respiratory Medicine Department, University Hospital of Larissa, Faculty of Medicine, University of Thessaly, 41500 Larissa, Greece; Paraskevi.kirgou@gmail.com (P.K.); kgourg@med.uth.gr (K.G.); 3 Bioinformatics Laboratory, Department of Biochemistry and Biotechnology, University of Thessaly, 41500 Larissa, Greece; amoutzias@bio.uth.gr; 4 Department of Biochemistry, Faculty of Medicine, University of Thessaly, 41500 Larissa, Greece

**Keywords:** lung cancer, exhaled breath, volatile organic compounds, machine learning

## Abstract

The aim of the present study was to investigate the ability of breath analysis to distinguish lung cancer (LC) patients from patients with other respiratory diseases and healthy people. The population sample consisted of 51 patients with confirmed LC, 38 patients with pathological computed tomography (CT) findings not diagnosed with LC, and 53 healthy controls. The concentrations of 19 volatile organic compounds (VOCs) were quantified in the exhaled breath of study participants by solid phase microextraction (SPME) of the VOCs and subsequent gas chromatography-mass spectrometry (GC-MS) analysis. Kruskal–Wallis and Mann–Whitney tests were used to identify significant differences between subgroups. Machine learning methods were used to determine the discriminant power of the method. Several compounds were found to differ significantly between LC patients and healthy controls. Strong associations were identified for 2-propanol, 1-propanol, toluene, ethylbenzene, and styrene (*p*-values < 0.001–0.006). These associations remained significant when ambient air concentrations were subtracted from breath concentrations. VOC levels were found to be affected by ambient air concentrations and a few by smoking status. The random forest machine learning algorithm achieved a correct classification of patients of 88.5% (area under the curve—AUC 0.94). However, none of the methods used achieved adequate discrimination between LC patients and patients with abnormal computed tomography (CT) findings. Biomarker sets, consisting mainly of the exogenous monoaromatic compounds and 1- and 2- propanol, adequately discriminated LC patients from healthy controls. The breath concentrations of these compounds may reflect the alterations in patient’s physiological and biochemical status and perhaps can be used as probes for the investigation of these statuses or normalization of patient-related factors in breath analysis.

## 1. Introduction

According to the World Health Organization (WHO), cancer is the second leading cause of death globally, exceeded only by heart disease, while lung cancer is the leading cause of all cancer-related deaths [1]. Moreover, cancer has the highest economic burden on society and individuals, and thus cancer prevention and control are nowadays the primary concern of medicine [2]. The fight against cancer is based on three main pillars that comprise development and application of: (1) disease prevention measures, (2) new methods for disease screening and early detection, and (3) new cancer treatment drugs and therapies. Early cancer detection by effective screening tests has substantially decreased death rates for breast cancer, melanoma, cervical cancer, and colorectal cancer [3,4]. Given these successful achievements and general acceptance of the concept that population preventive screening will reduce cancer mortality, there are currently great investments into the development of new effective screening tests and new screening strategies [5,6]. In this context, breath analysis is considered to have great potential. Human breath contains volatile organic compounds (VOC) that are distinguished as endogenous, related to the organism’s metabolic and biological processes, or exogenous, inhaled or ingested from the environment or originating from human microbiota bioactivities. In case of disease, the metabolic and biological pathway can be dysregulated or altered [7], and this will change the composition of exhaled breath in endogenous VOCs. This change can be detected and used for disease detection and diagnosis. This initial concept has recently been extended by the understanding that exogenous compounds in breath can also offer information about an organism’s biochemical and physiological status [8]. The absorption, distribution metabolism and excretion of the exogenous compounds can also be influenced by disease. Besides this strong biochemical rationale, breath analysis is very attractive for population screening as breath sampling is completely non-invasive, readily available and does not require patient transfer to hospitals and other health facilities.

However, despite its attractiveness, obvious advantages and exuberance of research activity in the field for about twenty years, breath tests are still not approved and applied in routine clinical practice for early cancer detection and preventive screening, e.g., for lung cancer with still high mortality associated with late diagnosis [9]. Several reasons can be indicated for this apparently low outcome resulting from several decades of research efforts. Firstly, most published studies describe the classification of patients with confirmed lung cancer from healthy controls [10,11]. However, to discriminate cancer from health is not sufficient for clinical test application because other respiratory diseases have similar symptoms and biochemical backgrounds and can thus confound early cancer diagnosis. For example, it has been demonstrated that acute and chronic lung diseases, including cancer, are associated with increased oxidative stress and formation of common oxidative stress biomarkers [12]. Therefore, the value of a new test is not only to distinguish cancer patients from healthy people, but also from patients with other lung diseases.

To achieve this goal, the search for distinctive biomarkers should be based not only on untargeted analytical approaches mainly applied previously, but also on investigation of the biochemical background of the pathophysiological processes that underlie the disease. The understanding of this background can contribute to the prediction and validation of the distinctive biomarkers for specific diseases. Until recently, the mechanism of endogenous VOC biomarkers origin in breath has been scarcely investigated and poorly understood [13].

Another point of concern in breath research is the methodological quality (design and conduct) of the breath test diagnostic accuracy studies, which is still to be substantially improved. Recent systematic reviews on breath tests application for cancer diagnosis demonstrate that the published studies have a high risk of bias in the estimations of test accuracy, sensitivity and specificity, mainly due to issues regarding patient selection, method validation and flow and timing issues [14,15]. The high risk of bias questions the detected cancer VOC biomarkers and delays breath test approval for clinical practice.

In the present study, we investigated the possibility of breath analysis to distinguish: (i) lung cancer patients from patients with other respiratory diseases, and (ii) lung cancer patients from healthy people on the basis of 19 breath VOCs, previously indicated as potential lung cancer biomarkers.

## 2. Results

### 2.1. Population Characteristics

From the 89 patients with pathological computed tomography (CT) findings who underwent bronchoscopy with transbronchial biopsy (TBBX) and/or endobronchial ultrasound-guided transbronchial needle aspiration (EBUS-TBNA), lung cancer was diagnosed in 51 patients, according to the results of the cytological/histological examinations. Gender, smoking habit, and body mass index for LC patients, patients without LC, and the control group are presented in Table 1. Patients and controls were of similar age. Body mass index was slightly higher in healthy volunteers compared to patients. In regard to smoking habit, most of the LC patients were former smokers, and only 5.9% reported that they had never smoked. Patients that were not diagnosed with LC had slightly different frequencies of smoking habit. In the control group, the percentage of active smokers was significantly higher, as was the percentage of individuals that had never smoked. Patients with lung issues seeking medical care are more likely to have quit smoking, presumably due to health problems, but as expected, the vast majority had smoked in the past. In Supplementary Table S1, we disclose data for each participant including age, gender, BMI, CT findings, and reported symptoms.

### 2.2. Breath Concentrations and Determinants of Investigated VOCs

**Figure 1:**
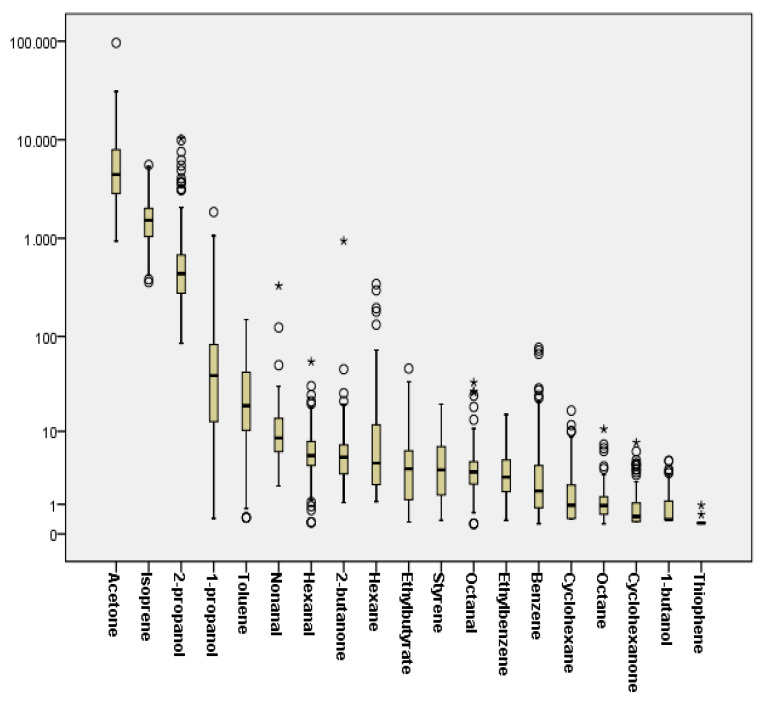
Median breath concentrations (ng/L air) in study participants. ο: outliers, *: extreme values

Most of the investigated compounds were detectable in the exhaled breath of study participants. In Supplementary Table S2, the detection frequencies for each compound are presented, along with the medians, interquartile range (IQR), and minimum and maximum concentrations. Some substances were detected in 100% of the participants, namely isoprene, acetone, 2-propanol, and nonanal, while the lowest detection frequencies were observed for thiophene (1.4%), 1-butanol (40.1%), and cyclohexanone (52.4%). In Figure 1, a summary of the median VOC concentration for each VOC is given. From the selected compounds, isoprene and acetone were the most abundant in the breath of the population examined. High concentrations of 2-propanol and 1-propanol were also detected. Regarding aromatic compounds, the highest concentrations were observed for toluene, while significantly lower levels were measured for benzene, styrene, and ethylbenzene. Aldehydes (hexanal, octanal, nonanal) were also present in the vast majority of breath samples analyzed.

Before assessing the ability of breath analysis to identify lung cancer, we investigated potential determinants of breath concentrations, and particularly smoking habit, ambient air VOC concentrations, age, BMI, and gender. In Table 2, we provide results regarding the correlation of each compound with the corresponding environmental air concentrations, age, gender, smoking status, and BMI. For the majority of VOCs, the correlation between ambient and exhaled air concentrations was strong, demonstrating the dependence of breath composition on environmental air composition. For some substances such as hexane, cyclohexane, and nonanal, correlation coefficients were notably high, explaining a large percentage of the variance. Differences were also observed in the concentrations of several VOCs between groups categorized by smoking status. Benzene median concentrations were approximately six times higher in smokers, while the concentrations of toluene, hexanal, and octane (data not shown) were also significantly higher. Styrene and ethylbenzene breath levels were also increased; however, the association was not statistically significant. Age was negatively correlated with 1-butanol, octane, ethylbenzene, and styrene. Body mass index and gender were not significantly associated with any of the investigated compounds.

### 2.3. Association of Exhaled Breath VOCs with Disease Status

In Table 3, the summary of the medians (IQR) for the investigated VOCs is presented for LC patients (labeled as Ca^+^), patients without LC diagnosis (labeled as Ca^−^), and healthy controls (labeled as HC). Since exhaled breath concentrations were found to be affected by the ambient air composition, the breath subtracts (ΔC = C_exhaled_ − C_ambient_) were also calculated for each compound investigated, by subtracting the concentration of ambient air from exhaled breath. Median (IQR) breath subtracts for each population group are also presented in Table 3**,** along with the percentage of samples with a positive subtract for each compound. Several compounds were found to differ significantly between LC patients and healthy controls. In particular, strong associations were identified for 2- propanol, 1-propanol, toluene, ethyl benzene, and styrene, while milder associations were found for cyclohexane and benzene. When breath subtracts were considered, the associations remained strong for toluene, ethylbenzene, styrene, and 1-propanol. For the abovementioned substances, the percentage of participants having a positive breath subtract (exhaling higher concentrations than inhaling) was different between patients and controls. When Ca^+^ patients were compared to Ca^−^ patients, no significant associations were found. Breath concentrations in direct comparison with ambient concentrations for each VOC are given in Supplementary Figures S1–S17. 

As previously reported (Section 2.2), smoking status was correlated with concentrations of several VOCs. To examine the potential confounding effect of smoking habit, we conducted comparative analysis to determine the differentiation of VOC levels between patient groups, stratified for smoking status. Table 4 presents the corresponding *p*-values (Mann–Whitney test) in active smokers and former/never smokers. It can be observed that the same compounds differ significantly in both strata, with the exceptions of 2-propanol and cyclohexane, where in the active smokers’ group, non-significant associations were found. The weaker associations observed in the active smokers’ stratum are likely due to the smaller number of participants. In general, the association of aromatic compounds and 1-propanol with disease was consistent in both smokers and non-smokers.

### 2.4. Application of Machine Learning Methods to Determine the Diagnostic Efficiency of the Breath Test

To determine the discriminatory power of breath analysis between LC patients (51 individuals) and the control group (53 individuals), we applied three widely used machine learning methods, specifically naïve Bayes, logistic regression, and random forests with 10-fold cross-validation in two major categories of datasets, based on: (i) exhaled breath VOC concentrations (C_exhaled_), or (ii) breath subtract concentrations (ΔC = C_exhaled_ − C_ambient_). Each of these two categories contained three different sets of compounds. The first set contained all 19 VOCs. The second set contained a select subset of VOCs, which were identified as statistically significant from the Mann–Whitney test for exhaled breath or breath subtract (Table 3). The third set contained compounds identified as informative by two or more selection methods (out of four mentioned in Materials and Methods from Weka and the Mann–Whitney tests). Thus, when breath concentrations were considered, the reduced datasets contained seven informative VOCs (identified from the Mann–Whitney test—Table 3) and six VOCs that were identified by two or more selection methods, namely 2-propanol, hexane, 1-propanol, toluene, ethyl-benzene, and styrene.

Respectively, when breath subtracts were used, the reduced datasets contained seven informative VOCs (identified from the Mann–Whitney test—Table 3) and five compounds identified by two or more selection methods, namely 1-propanol, toluene, ethyl-butyrate, ethyl-benzene, and styrene. As evident from Table 5, discrimination between cancer patients and controls was satisfactory with the correct classification of data points, ranging between 76 and 89% for the best performing algorithm of random forests, whether breath measurements or the breath subtracts were used. The same conclusions hold even if the data are stratified into smokers and non-smokers. The best performance was observed for the discrimination of Ca^+^ from HC, using breath concentrations of all 19 VOCs, with the random forest algorithm achieving a correct classification for 88.5% of the data points. Overall, the highest performing algorithm was random forest; however, logistic regression also performed very satisfactorily. The discrimination was found to be very satisfactory (83.7%) even when only seven VOCs were included in the analysis. The use of only five metabolites in the subtract dataset allowed the algorithm to correctly classify 79% of Ca^+^ and HC. We further tested the potential for discrimination between cancer patients (51 individuals) vs. non-cancer patients (38 individuals), with the three machine learning algorithms. As evident from Table 5, the set of 19 VOCs was not capable of discriminating between cancer and non-cancer patients, irrespective of the machine learning algorithm applied: either we used raw breath or subtract measurements. Again, the same conclusions hold even if the data are stratified into smokers and non-smokers. Furthermore, we did not identify informative features for this type of classification, in accordance with the lack of statistically significant differences from the Mann–Whitney test.

Next, we tested the possibility of using the 19 VOCs to discriminate non-cancer patients (38 individuals) from the control group (53 individuals), for both raw breath measurements and for subtracts. Once again, random forests managed to correctly classify 82% and 80% of data points for raw breath and subtracts, respectively. This observation was expected given the very good discrimination between cancer patients and controls, as well as the lack of discrimination between cancer and non-cancer patients. Finally, we merged the cancer and non-cancer patients into one disease group (with 89 individuals) and compared it against the control group (53 individuals). We thus generated a dataset for raw breath and a dataset for breath subtracts. Both logistic regression and random forests managed to correctly classify 81–83% of the data points. We therefore concluded that the given set of compounds has sufficient discriminatory power to help us understand if a sample originates from a healthy person or from a person with a pulmonary disease. However, it is not possible to discriminate cancer from non-cancer patients. Results regarding the discrimination of Ca^−^ from HC, and all patients from HC are given in Supplementary Table S3.

In our analyses, we observed that the set of all 19 metabolites had a slightly better discriminatory power than the various other subsets of informative metabolites that we identified. We hypothesize that this may be attributed to the fact that some of the metabolites that were not included within the various subsets may still hold some signal/information, albeit weak.

## 3. Discussion

In our study, we investigated variations in the concentrations of 19 VOCs in breath samples of patients with confirmed lung cancer (Ca^+^), of patients with abnormal CT where lung cancer was not confirmed (Ca^−^), and that of healthy controls (HC). We also investigated if these three groups could be differentiated based on the identified variations. The 19 VOCs selected have often been referred to as lung cancer biomarkers [14,15,16,17]. In this study, we identified several compounds with significantly differentiated breath concentrations in LC patients compared with healthy controls. Different combinations of these biomarkers achieved an adequate classification of LC patients and healthy controls but failed to distinguish LC patients from patients with abnormal CT findings.

Numerous compounds have been detected in the breath of healthy and diseased people [18] and the profile of these compounds depends on various determinants such as sampling method, sampling environmental, patient-related factors, as well as the analytical approach used. In spite of its complexity, breath analysis demonstrates high accuracy of discrimination between cancer patients and healthy controls, with a mean area under the receiver operating characteristic analysis curve of 0.94 (SE 0.01) for 63 recently reviewed studies, pooled sensitivity of 79% (95% CI, 77–81%), and specificity of 89% (95% CI, 88–90%) for the same studies [14]: this is comparable or better than diagnostic efficiency of current lung cancer screening methods [15,19]. In another recent review, Hua et al. summarized studies about exhaled VOCs for LC screening, reporting sensitivities ranging from 60.6% to 100% and specificities ranging from 41% to 100%, respectively [15]. Results of our study demonstrate that 19 compounds successfully classify Ca^+^ vs. HC, Ca^−^ vs. HC, and all patients (Ca^+^ and Ca^−^) vs. HC with high accuracy and high discrimination ability (accuracy 89%, 82%, and 82% and discrimination ability expressed as AUC 0.940, 0.906, and 0.948, respectively, Table 5, Supplementary Table S3). Our data reveal that from 19 compounds measured, seven differed significantly in their concentrations in exhaled breath samples from LC patients and controls, namely 2-propanol, cyclohexane, 1-propanol, benzene, toluene, ethylbenzene, and styrene. The reduction of variables from 19 to these 7 compounds slightly reduced the discrimination of Ca^+^ vs. HC to 84% accuracy (from 89%) and 0.908 AUC (from 0.940) (Table 5). The reduction of variables can substantially reduce the time of GC-MS analysis and increase the method throughput in future clinical applications. It is interesting that among these seven most significant variables, there are four monoaromatic compounds (toluene, ethylbenzene, styrene, and benzene). However, the mechanism that could lead to increased breath concentrations still remains unknown, and the endogenous generation of these compounds as results of human metabolism is considered unlikely. These compounds are known as significant environmental pollutants and health hazards arising from natural sources and anthropogenic activities [20]; cigarette smoking is also considered as an important cause of human exposure [21]. However, confounding effects of smoking in associations found through our study are unlikely, since non-smokers Ca^+^ can be successfully discriminated from non-smoker controls using the seven abovementioned variables (Table 5, non-smokers, seven variables). Several studies in the scientific literature have associated breath concentrations of monoaromatic compounds with LC [22,23,24,25]. After exposure and inhalation, the inhaled compounds enter the systematic circulation and are transferred to different body compartments, where they can be deposited or metabolized in the liver. Afterwards, the metabolite and their unmetabolized precursors are excreted, and it is accepted that VOC are mainly excreted by exhalation [26,27]. The exhaled concentration of exogenous compounds such as toluene depends on the inhaled concentration as well as lung ventilation rate, metabolism rate, and other physiological parameters (e.g., cardiac output). Impaired pulmonary function (pulmonary ventilation and capacity) in lung cancer and in benign pulmonary diseases obviously reduces the amount of exogenous compounds inhaled (but not concentration), the level of circulation transfer, and as a consequence, increases the metabolic ratio (e.g., the ratio of parent compound/metabolite). Therefore, increased concentrations of unmetabolized exogeneous VOC can be detected in exhaled breath and this can partially explain the significantly increased concentrations of toluene, ethylbenzene, and styrene observed in the exhaled breath of patients (Ca^+^ and Ca^−^), compared to healthy controls. We can thus suppose that the observed variations in the concentrations of monoaromatic compounds may reflect alterations in pulmonary function, or more broadly, the physiological and biochemical status of the patient. However, this model cannot explain the fact that patients are the source of these three monoaromatic compounds with median ΔC = C_exhaled_ − C_ambient_ > 0. ΔC may have values ΔC > 0 when the respiratory system is the source of the compound, ΔC < 0 when the respiratory is the sink, and C = 0 when C exhaled = C ambient [28]. The ΔC value depends on the VOC ambient concentration and its physiologically based pharmacokinetics that comprise compound deposition in adipose tissue and metabolism in the liver [26,29]. The compound deposition/release rate in adipose tissue depends on tissue volume [29,30]. Loss of adipose and muscle tissue is often observed in cancer, COPD, pulmonary fibrosis, and cachexia [31]. These changes in body composition can lead to increased release of deposited compounds and, in our case, of toluene, ethylbenzene, and styrene. It is known that following weight loss, lipophilic organic pollutants are released from adipose tissue into the blood circulation and their increased levels can be detected for a long period [32,33,34]. This hypothesis also agrees with our observation that increased levels of monoaromatic compounds are observed in the breath of Ca^+^ and Ca^−^ patients vs. HC in spite of the fact that the concentrations of these compounds were significantly lower in the ambient air of LC sampling in comparison to that for HC sampling room (Supplementary Figures S9, S13, and S14). Benzene with median ΔC close to 0 is an exception in the analyzed group of monoaromatic compounds. Moreover, this compound was not indicated as informative by the feature selection methods.

The aliphatic alcohols, 2-propanol and 1-propanol also proved to be significant determinants for the discrimination of Ca^+^ and Ca^−^ from healthy controls. These alcohols in human breath can be of endo- or exogenous origin, either from metabolic processes (acetone reduction, amino acid breakdown) or from exposure to 2-propanol and 1-propanol from external sources, e.g., from disinfectants widely used in health care units [35]. In case of exposure, alcohols are rapidly absorbed and metabolized (reversibly) to corresponding aldehydes—acetone and propanal. This can explain the negative breath subtract (ΔC < 0) observed in most patients (Ca^+^ and Ca^−^) and healthy controls. The reaction is catalyzed by alcohol dehydrogenase (ADH) that can catalyze both direct and reverse reactions, and under certain conditions, accelerate the reduction of acetone to 2-propanol [36,37]. ADH is expressed mostly in the liver but also can be found in the lungs, kidneys, and gastrointestinal tract (duodenum and colon). In tissues of many cancers, significantly higher activity of total alcohol dehydrogenase has been observed [38], which can perhaps explain lower concentrations of 2-propanol and 1-propanol in the breath subtracts of Ca^+^ and Ca^−^ patients in comparison to healthy controls. Positive breath subtracts for 2-propanol and 1-propanol have been observed more frequently (detected) in healthy controls than in patients (Table 3: 43.4, 21.6, and 15.8% correspondingly for 2-propanol and 35.8, 11.8, and 21.0% for 1-propanol). The increased activity of ADH in cancer can probably explain our observation that in spite of the fact that ambient median concentrations of isopropanol for LC patients were more than 3-fold higher compared to these of HC (750.4 ng/L vs. 242.9 ng/L air), the difference in breath isopropanol was less than one and a half (527.7 ng/L air vs. 315.5 ng/L air) (Supplementary Figure S3).

Isoprene and acetone, which were the most abundant compounds in the breath of the study participants, are known to exist in every person’s breath [39]. Acetone is a product of lipolysis, ketogenic amino-acid breakdown, and oxidation of 2-propanol [36], while isoprene is constantly being produced endogenously and is linked to cholesterologenesis [40]. Acetone and isoprene breath levels did not differ significantly between cancer patients and controls, although some previous publications have indicated these substances as potential biomarkers for LC [24,41,42,43,44]. Cyclohexane breath levels were higher in healthy controls, but the association should be interpreted carefully due to the strong correlation between ambient and exhaled air concentrations (Spearman r = 0.730). Aldehydes, although frequently detected, showed similar concentrations in patients and controls. The sources of aldehydes in exhaled breath can be both exogenous (personal care products, food additives) and endogenous (formed during fatty acid peroxidation) [45].

In general, compounds identified by our analysis have been previously reported as potential biomarkers. However there were several VOCs reported in previous studies that were not found to differ significantly between patients and controls, such as aldehydes, isoprene, and acetone, among others. To advance the understanding of exhaled breath patterns and their relation to lung cancer or other pathological conditions, this lack of reproducibility should be interpreted, exploring the array of uncertainties and potential confounding factors. Standardization of sampling, storage, and analysis has been proposed as a measure for mitigating heterogeneity, but for the moment there are no (to our knowledge) multicenter studies reporting reproducible results. Another common obstacle is the lack of understanding of biochemical processes reflected in VOC breath profiles.

Comparative analysis between Ca^+^ patients and Ca^−^ patients did not reveal statistically significant differentiation for any of the VOCs analyzed. The comparison group of bronchoscopy patients recruited in this study consisted of patients suspected to have LC due to CT findings, but the presence of cancer was not confirmed by histological/cytological examinations. The lack of discriminant power in distinguishing LC patients from patients not diagnosed with LC raises some concerns regarding the potential use of exhaled breath analysis as a diagnostic tool. The literature suggests that there is an overlap in discriminative VOCs in pulmonary diseases and disease-specific biomarkers are needed [46]. The vast majority of past studies adopted a case–control design, comparing LC patients only with healthy individuals; they had not assessed the performance of exhaled breath analysis in discriminating LC from other serious benign pulmonary conditions [15]. The inability to discriminate these diseases from LC will obviously reduce the potential use of the method in clinical applications. Concerning Ca^+^ and Ca^−^ patients, it can be supposed that selected biomarkers have common endogenous or exogenous origin in benign pulmonary diseases and cancer. Thus, the search for specific VOC to discriminate these two groups should be the objective of future research. The ability of breath analysis to distinguish lung cancer patients from patients with benign conditions has been less studied. One recent publication compared LC patients to patients with benign pulmonary diseases (chronic obstructive pulmonary disease (COPD), asthma, pneumonia, pulmonary embolism, and benign lung tumors) [47]. Similarly to our study, the authors reported adequate discrimination between LC patients and healthy controls, but state that LC and benign tumor subjects were hardly distinguishable. Somewhat better results were reported from a study that analyzed breath samples from LC patients and benign pulmonary nodules, by Fourier transform ion cyclotron resonance mass spectrometry (FT-ICR-MS). Authors report 100% sensitivity but relatively low specificity (64%) [48]. To mitigate the risk of bias and support the applicability of breath analysis in cancer diagnosis, further research is needed to discover not only the biomarkers that discriminate LC patients from healthy individuals, but also a group of cancer-specific biomarkers able to distinguish LC patients from patients with other pulmonary pathological findings similar to those observed in lung cancer. Another potential application of breath analysis could involve the use of breath tests as pre-screening tools preceding CT screening [16].

## 4. Materials and Methods

### 4.1. Experimental

#### 4.1.1. Reagents and Materials

Analytical standards for 19 volatile organic compounds were purchased from Sigma-Aldrich (St. Louis, MO, USA). Air sampling bags (1 L Tedlar^®^ PLV Gas Sampling Bag w/Thermogreen^®^ LB-2 Septa) were also acquired from Sigma-Aldrich. For the isolation and pre-concentration of the analytes, a solid phase microextraction (SPME) fiber assembly, Carboxen/Polydimethylsiloxane (CAR/PDMS, df 75 µm, acquired from Supelco/Sigma-Aldrich, was used, incorporated in an SPME fiber manual holder (Supelco/Sigma-Aldrich).

#### 4.1.2. Breath Sampling

Breath samples were collected in Tedlar^®^ bags. Before use, Tedlar^®^ bags were flushed with pure nitrogen to remove any contamination. Three consecutive flushes were conducted for each bag. For the collection of exhaled breath, participants were asked to inhale deeply and hold their breath for 30 s, then exhale through a disposable mouthpiece into the 1 L Tedlar^®^ bag until filled. The procedure was repeated; two breath samples were collected from each volunteer. The collection of two samples was performed to reduce breath-to-breath variations and the process of breath holding to reduce non-homogeneity of air samples and enhance the reproducibility of measurements [49,50].

Breath samples were collected with approximately two-minute intervals in between. Ambient air samples were also collected with the use of a portable Laboport^®^ UN 86 KTP (KNF Neuberger GmbH, Freiburg, Germany) pump. A rubber hose was attached to the pump’s outlet and in the tedlar bag’s valve. The flow rate for air sampling was 5.5 L/min. Samples were analyzed within 6 h. Individuals who agreed to participate were moved to a room prepared for breath sampling. The room was designated by the hospital or health center personnel. At the days of sampling, no other routine tasks were performed in the sampling area which was accessible only to the researchers. Breath sampling from all participants was not conducted in the same room. Used Tedlar^®^ bags were analyzed for residual contamination. Some VOCs were detected in used bags, but after 3 consequent flushes with pure nitrogen, the concentrations of the analyses were below the LOD, with the exception of acetone, where traces slightly above the method LOD were observed after flushing. Tedlar^®^ bags were re-used for no more than 5 samples.

#### 4.1.3. Solid Phase Microextraction

Extraction and pre-concentration of the analytes from breath samples was achieved by solid phase microextraction (SPME) using a 75 μm Carboxen-polydimethylsiloxane (CAR/PDMS)-coated fused silica fiber assembly incorporated into an SPME Manual Holder. Prior to application, SPME fibers were conditioned in GC inlet for 30 min at 250 °C. A blank analysis was conducted to assure that the fiber was clean, and no extraneous peaks were present on the blank chromatogram. Afterwards, the fiber was introduced into the sampling bag by piercing the septum and was exposed to the breath sample for 25 min at room temperature. After extraction, the fiber was inserted into GC inlet. Desorption of analytes from the fiber was performed for 5 min at 270 °C. The efficiency of SPME and fiber performance was followed throughout the study and estimated by the level of standards’ peak area in the calibration graph prepared daily and the linearity of the instrument response. The same fiber was used for the instrument calibration and samples analysis to avoid possible intra- and interlot sorption variabilities. The fiber was changed after approximately 100 injections. The effect of humidity, solvent, and matrix VOCs on SPME was considered negligible.

#### 4.1.4. GC-MS Analysis

A Finnigan Trace GC Ultra/Polaris Q Quadrupole Ion Trap GC/MSn system was used for the quantification of analytes in breath samples. The gas chromatograph was equipped with a programmed temperature vaporizing injector (BEST PTV, Thermo Electron Corporation, Waltham, MA, USA) and a DB-624 GC Column capillary column (Inner Diameter: 0.25 mm, Length: 30 m, Film: 1.4 μm, 6% Cyanopropylphenyl/94% Dimethylpolysiloxan, Agilent, Santa Clara, CA, USA). Helium was used as the carrier gas in constant flow mode at 2 mL/min. The PTV injector temperature was set to 270 °C and injections were made in splitless mode. The GC oven temperature program had an initial temperature of 40 °C for 5 min, and then ramped to 80 °C with a heating rate of 8 °C/min, then the heating rate changed to 30 °C/min until 190 °C, where it was held for 5 min. GC–MS chromatograms were acquired in TIC (total ion current), mode of mass analyzer, and then extracted at one or two specific *m*/*z* value for analyte quantification. Data acquisition and processing was carried out using the Xcalibur™ 3.0 software (Themo Fisher Scientific, Waltham, MA, USA). Mass range was set to 40–200 *m*/*z*.

#### 4.1.5. Evaluation of Analytical Method Performance

The method performance was evaluated in terms of linearity of detector response, limit of detection (LOD) and limit of quantitation (LOQ), accuracy, and precision. To investigate the linearity of the detector response (peak area), calibration graphs were generated with seven calibration concentration levels. The calibration standard concentrations covered the expected analyte concentration ranges in human breath. For the preparation of calibration standards, Tedlar^®^ bags were filled with high-purity nitrogen gas. Eight calibration solutions with known concentrations were prepared in methanol in 2 mL screw tap glass vials. For the preparation of spiked air mixtures, the septum of the bag was pierced by a 100 μL glass micro-syringe and infused with 10 μL of the corresponding calibration solution. A blank air mixture was also prepared by injecting 10 μL of methanol into the bag. The samples were allowed to balance for 1 h. Detection and quantification limits were calculated from the standard error (s) and the slope of the curve. The range of calibration, detection limits (LOD), quantification limits (LOQ), and the linearity of the detector response (R2) are presented in Supplementary Table S4. The method demonstrates good linearity over a range of concentrations of two orders of magnitude. The inter-day variations of calibration curve parameters (slope, intercept, and linearity) were evaluated by calibrations graphs generation within five consecutive days (Supplementary Table S4).

### 4.2. Study Participants Recruitment

The study population consisted of 89 patients from the General University Hospital of Larissa (Greece) who were scheduled for bronchoscopy due to abnormal CT findings. A group of 53 healthy individuals of similar age were recruited from local health centers as a control group. Samples were collected from October 2018 to October 2019. All participants were informed about the study and provided a signed consent form. After bronchoscopy, the course of the patients’ diagnosis was monitored prospectively and they were categorized according to the presence of LC, according to results of the cytological/histological examination. Thus, a new comparison group of bronchoscopy patients was formed (Ca^−^), consisting of patients suspected to have LC due to CT findings, however, the presence of cancer was not confirmed by histological/cytological examinations. The majority of these patients presented radiographic abnormalities such as lung infiltrates and masses, enlarged mediastinal hilar lymph nodes, and endobronchial lesions. Lung shadows as shown in a CT scan or a pulmonary symptom are not always indications of malignancies. Some possible causes include sarcoidosis, hypersensitivity pneumonitis, interstitial lung diseases, lymphoma, or pulmonary infections such as tuberculosis. Demographic characteristics, habits, and medical history were recorded with the use of questionnaires.

The study protocol was approved by the Scientific Council of General University Hospital of Larissa with the 16/20/06-12-2018 decision.

### 4.3. Statistical Analysis and Machine Learning Methods

The statistical analysis was performed by using the IBM SPSS Statistics V22.0 software. The mean concentration from the two samples acquired per participant was calculated to reduce variability. The normality of the distribution of quantitative variables was determined by the Kolmogorov–Smyrnof test. For the quantitative variables, the median value and the corresponding interquartile range (IQR) are presented. Kruskal–Wallis (>2 groups) and Mann–Whitney criteria were used to determine statistically significant differences between subgroups. Spearman correlation coefficients were calculated to investigate correlations between quantitative variables. Results with *p*-value <0.05 were considered statistically significant. For the analysis, analytes concentrations below the limit of detection were replaced with the LOD/sqtr2. When breath subtract concentrations were analyzed, negative values were replaced with zero.

The Waikato Environment for Knowledge Analysis (Weka) was used for machine learning analyses. In particular, the filtered data from each group were analyzed using naïve Bayes, logistic regression, and random forest methods, with 10-fold cross-validation. Detection of potentially informative metabolites that can separate the groups from each other was performed with the feature selection module within Weka, by using the following methods and their default parameters: “CorrelationAttributeEval, InfoGainAttributeEval, ClassifireAttributeEval, CfsSubSetEval”. In addition, we took into account the metabolites/features that were statistically different between the various groups, based on the previously mentioned Mann–Whitney test.

## 5. Conclusions

Exhaled breath analysis distinguished lung cancer patients from healthy controls with adequate accuracy. The discrimination is defined mainly on concentration variations of monocyclic aromatic VOCs ethylbenzene, toluene, styrene, and 2- and 1-propanol. However, discrimination of lung cancer patients from patients suspected to have lung cancer (but eventually not diagnosed) was inadequate. While we provide evidence that exhaled breath composition is associated with pulmonary pathological conditions, its use as a diagnostic tool needs to be supported by cancer-specific reproducible biomarker sets. We also formulate the hypothesis that in lung diseases, alterations in lung function and in absorption, metabolism, and excretion mechanisms of inhaled exogenous VOCs can be reflected in the exhaled breath VOCs profiles. Thus, the impact of these underlying mechanisms should be further investigated.

## Figures and Tables

**Table 1:** Characteristics of the study population.

		Patients Ca^+^		Patients Ca^−^		HC		*p*-Value *
		Mean (SD)		Mean (SD)		Mean (SD)		
Age		70.9 (8.1)		65.2 (13.2)		66.8 (10.7)		0.055
BMI		26.48 (4.37)		27.41 (4.72)		27.97(5.43)		0.365

		n	%	n	%	n	%	
Smoking status	Active smoker (daily)	7	13.7%	8	21.1%	20	37.7%	
	Active smoker (occasionally)	1	2.0%	2	5.3%	0	0.0%	
	Former smoker	40	78.4%	21	55.3%	18	34.0%	
	Never	3	5.9%	7	18.4%	15	28.3%	

Gender	Male	44	86.3%	31	81.6%	36	67.9%	

	Female	7	13.7%	7	18.4%	17	32.1%	
Total		51	100%	38	100%	53	100%	

Ca^+^: patients diagnosed with lung cancer, Ca^−^: patients with pathological CT findings not diagnosed with lung cancer by histological/cytological examination, HC: healthy controls, * Kruskal–Wallis test.

**Table 2:** Determinants of volatile organic compounds (VOC) breath concentrations.

	Gender	Smoking Status *	Ambient Air Concentrations	Body Mass Index	Age
	*p*-Value (Mann–Whitney Test)	Trend/*p*-Value (Mann–Whitney Test)	Correlation Coefficient **/*p*-Value	Correlation Coefficient **/*p*-Value	Correlation Coefficient **/*p*-Value
isoprene	0.575	↑/0.646	0.117/0.058	−0.021/0.800	−0.092/0.260
acetone	0.272	↓/0.246	0.044/0.585	−0.129/0.111	0.138/0.088
2-propanol	0.994	↓/0.332	0.383/<0.001	0.009/0.914	−0.067/0.408
hexane	0.676	↑/0.640	0.689/<0.001	0.007/0.993	−0.095/0.241
1-propanol	0.803	↓/0.827	0.545/<0.001	−0.061/0.453	−0.024/0.768
2-butanone	0.134	↑/0.080	0.176/0.029	−0.086/0.289	−0.068/0.406
cyclohexane	0.653	↑/0.702	0.730/<0.001	−0.001/0.987	0.049/0.549
benzene	0.834	↑/<0.001	0.416/<0.001	−0.076/0.350	−0.131/0.107
1-butanol	0.241	↑/0.752	0.347/<0.001	−0.050/0.537	−0.198/0.018
toluene	0.178	↑/0.007	0.131/0.106	−0.056/0.494	−0.135/0.095
octane	0.280	↑/0.005	0.200/0.013	0.069/0.394	−0.166/0.040
ethyl butyrate	0.238	↓/0.643	0.401/<0.001	−0.019/0.813	−0.125/0.125
hexanal	0.739	↑/< 0.001	0.332/<0.001	−0.039/0.613	−0.004/0.964
ethylbenzene	0.639	↑/0.104	0.253/0.002	−0.095/0.241	−0.177/0.029
styrene	0.334	↑/0.148	−0.160/0.048	−0.027/0.927	−0.240/0.003
cyclohexanone	0.997	↑/0.500	0.273/<0.001	−0.078/0.340	−0.033/0.690
n-octanal	0.931	↓/0.82	0.468/<0.001	−0.007/0.927	−0.063/0.441
nonanal	0.990	↑/0.187	0.731/<0.001	−0.034/0.674	0.006/0.940

* Active smokers vs. former/never smokers. ** Spearman correlation coefficients for all participants grouped together. ↑ smokers had higher concentrations, ↓ smokers had lower concentrations. Statistically significant associations are highlighted in bold.

**Table 3:** Concentrations of VOCs biomarkers (ng/L) in breath before and after ambient air correction and significance of their differences between investigated groups.

		Patients Ca^+^		Patients Ca^−^		Healthy Contrοls		Ca^+^ vs. HC	Ca^+^ vsCa^−^	All Groups
Substance		%†	Median(IQR)	%†	Median(IQR)	%†	Median(IQR)	*p* *	*p* *	*p* **
Isoprene	Br	100%	1486(1037–1986)	100%	1661(1123–2578)	100%	1493(1029–1952)	0.909	0.328	0.501
	Sbtr		1471(1020–1962)		1643(1099–2560)		1459(1011–1936)	0.843	0.332	0.481
Acetone	Br	100%	4565(3157–7921)	100%	3944(2839–5877)	100%	4303(2761–9580)	0.997	0.236	0.442
	Sbtr		4463(3090–7820)		3844(2763–5859)		4239(2697–9530)	0.982	0.229	0.412
2-propanol	Br	21.6%	528(324–804)	15.8%	490(382–702)	43.4%	315(218–497)	**0.002**	0.636	**0.002**
	Sbtr		neg.(neg.-neg.)		neg.(neg.-neg.)		neg.(neg.−130.97)	**0.041**	0.491	**0.015**
Hexane	Br	25.5%	4.43(1.13–24.48)	28.9%	9.63(3.25–36.05)	52.8%	3.42(2.25–5.19)	0.239	0.121	**0.007**
	Sbtr		neg.(neg.−0.05)		neg.(neg.−0.73)		0.11(neg.−1.45)	**0.006**	0.504	**0.022**
1-propanol	Br	11.8%	30.78(7.14–57.81)	21.0%	24.13(8.14–60.85)	35.8%	63.84(38.46–103.63)	**<0.001**	0.684	**<0.001**
	Sbtr		neg.(neg.-neg.)		neg.(neg.-neg.)		neg.(neg.−14.09)	**0.005**	0.255	**0.014**
2-butanone	Br	82.3%	4.39(3.03–6.9)	92.1%	4.71(3.41–6.78)	92.4%	5.25(3.27–7)	0.358	0.507	0.626
	Sbtr		2.68(1.08–5.31)		2.98(1.39–5.31)		3.28(1.77–5.04)	0.321	0.531	0.576
Cyclohexane	Br	29.4%	0.69(0.43–2)	36.8%	0.92(0.61–1.99)	39.6%	1.46(0.43–2.48)	**0.050**	0.165	0.115
	Sbtr		neg.(neg.−0.22)		neg.(neg.−0.44)		neg.(neg.−0.49)	0.415	0.492	0.681
Benzene	Br	49.0%	1.33(0.66–3.17)	60.5%	1.63(0.88–3.23)	45.3%	2.42(1.21–5.15)	**0.028**	0.156	0.072
	Sbtr		neg.(neg.−0.79)		0.19(neg.−1.54)		neg.(neg.−3.68)	0.615	0.254	0.54
Thiophene ***	Br	0.0%	Nd	2.6%	nd	1.9%	nd	-	-	-
	Sbtr		Nd		nd		nd	-	-	-
1-butanol	Br	19.6%	nd(nd−1.05)	28.9%	nd(nd−1.07)	18.9%	nd(nd−1.41)	0.42	0.575	0.707
	Sbtr		neg.(neg.-neg.)		neg.(neg.−0.1)		neg.(neg.-neg.)	0.865	0.282	0.51
Toluene	Br	86.2%	27.36(15.35–66.04)	76.3%	24.39(18.14–51.17)	58.5%	12.33(6.27–21.37)	**<0.001**	0.816	**<0.001**
	Sbtr		18.16(2.32–56.58)		18.34(0.95–48.08)		0.87(neg.−4.21)	**<0.001**	0.592	**<0.001**
Octane	Br	74.5%	0.81(0.5–1.38)	73.7%	0.88(0.55–1.5)	77.3%	0.99(0.75–1.33)	0.09	0.871	0.186
	Sbtr		0.42(neg.−0.86)		0.32(neg.−0.71)		0.36(0.06–0.76)	0.883	0.907	0.965
Ethyl butyrate	Br	58.8%	3.11(0.84–6.03)	52.6%	2.08(0.52–4.95)	79.2%	4.28(2.49–6.24)	0.085	0.221	**0.015**
	Sbtr		0.9(neg.−3.11)		0.26(neg−3.45)		2.49(0.53–3.9)	**0.021**	0.461	**0.010**
Hexanal	Br	29.4%	5.13(3.43–6.95)	28.9%	5.24(4.35–7.2)	22.6%	5.36(4.04–10.76)	0.172	0.275	0.313
	Sbtr		neg.(neg.−0.33)		Neg.(neg.−1.17)		neg.(neg.-neg.)	0.634	0.918	0.856
Ethyl_benzene	Br	76.4%	3.85(2.44–6.26)	76.3%	3.13(2.02–5.36)	49.0%	2.01(1.30–2.89)	**<0.001**	0.476	**<0.001**
	Sbtr		1.99(0.18–4.15)		2.05(0.06–3.56)		neg.(neg.−1.78)	**<0.001**	0.442	**<0.001**
Styrene	Br	86.2%	4.83(2.36–7.87)	89.5%	4.85(1.71–8.26)	62.3%	2.11(1.25–3.53)	**<0.001**	0.914	**<0.001**
	Sbtr		4.02(0.53–6.93)		3.97(0.62–7.48)		0.28(neg.−1.57)	**<0.001**	0.888	**<0.001**
Cyclohexanone	Br	39.2%	0.58(nd−1.04)	42.1%	nd(nd−1.53)	39.6%	0.49(0.34–0.92)	0.466	0.875	0.799
	Sbtr		neg.(neg.−0.36)		neg.(neg.−0.81)		neg.(neg.−0.28)	0.687	0.513	0.579
Octanal	Br	19.6%	3.1(1.65–4.99)	26.3%	2.97(2.06–3.90)	13.2%	3.46(2.66–4.44)	0.354	0.947	0.416
	Sbtr		neg.(neg.-neg.)		neg.(neg.−0.11)		neg.(neg.-neg.)	0.464	0.323	0.287
Nonanal	Br	7.8%	9.3(5.72–16.03)	7.9%	7.71(6.02–11.71)	0.0%	8.61(6.15–13.38)	0.407	0.131	0.317
	Sbtr		neg.(neg.-neg.)		neg.(neg.-neg.)		neg.(neg.-neg.)	0.117	0.993	0.114

Br: corresponds to breath concentration, Sbtr: corresponds to breath subtract concentrations, %† percentage with a positive breath subtract Ca^+^: patients diagnosed with lung cancer, Ca^−^: patients with pathological CT findings not diagnosed with lung cancer by histological/cytological examination, HC: healthy controls. * Mann–Whitney test. ** Kruskal–Wallis test. nd: not detected, neg.: subtract < 0. *** *p*-values not calculated due to very low detection frequency. Statistically significant associations are highlighted in bold.

**Table 4:** Comparative analysis to determine associations between VOC breath concentrations and disease status, stratified by smoking habit.

		Ca^+^ vs. HC (*p*-Value) *		Ca^+^ vs. Ca^−^ (*p*-Value) *	
Substance	Variable	Active Smokers	Former/Never Smokers	Active Smokers	Former/Never smokers
Isoprene	Br	0.601	0.882	0.068	0.787
	Sbtr	0.636	0.746	0.068	0.805
Acetone	Br	0.862	0.674	0.965	0.300
	Sbtr	0.862	0.694	0.965	0.290
2-propanol	Br	0.199	0.021	0.672	0.751
	Sbtr	0.746	0.011	0.829	0.635
Hexane	Br	0.182	0.336	0.762	0.170
	Sbtr	0.123	0.039	0.122	0.520
1-propanol	Br	0.003	0.002	0.460	0.621
	Sbtr	0.409	0.008	0.460	0.534
2-butanone	Br	1.000	0.486	0.762	0.502
	Sbtr	0.940	0.495	0.897	0.945
Cyclohexane	Br	0.469	0.007	0.696	0.088
	Sbtr	0.553	0.226	0.829	0.635
Benzene	Br	0.746	0.404	1.000	0.225
	Sbtr	0.746	0.079	0.629	0.918
1-butanol	Br	0.901	0.294	0.897	0.844
	Sbtr	0.636	0.672	0.897	0.352
Toluene	Br	0.043	<0.001	0.515	0.724
	Sbtr	0.033	<0.001	0.633	0.435
Octane	Br	1.000	0.253	0.829	0.911
	Sbtr	0.566	0.664	0.762	0.725
Ethyl butyrate	Br	0.237	0.255	0.630	0.540
	Sbtr	0.033	0.252	0.897	0.526
Hexanal	Br	0.940	0.408	0.696	0.685
	Sbtr	0.438	0.580	0.897	0.510
Ethyl_benzene	Br	0.011	<0.001	1.000	0.638
	Sbtr	0.018	<0.001	0.762	0.538
Styrene	Br	0.079	<0.001	0.274	0.742
	Sbtr	0.028	<0.001	0.315	0.671
Cyclohexanone	Br	0.258	0.734	0.696	0.955
	Sbtr	0.601	0.785	1.000	0.642
Octanal	Br	0.150	0.904	0.762	0.944
	Sbtr	0.940	0.284	0.315	0.882
Nonanal	Br	0.381	0.175	0.696	0.066
	Sbtr	0.636	0.124	0.762	0.536

Ca^+^: patients diagnosed with lung cancer, Ca^−^: patients with pathological CT findings not diagnosed with lung cancer by histological/cytological examination, HC: healthy controls, Br: corresponds to breath concentration, Sbtr: corresponds to breath subtract concentrations. * Mann–Whitney test. Statistically significant associations are highlighted in bold.

**Table 5:** Summary of correct classification, based on naïve Bayes, logistic regression, and random forest machine learning methods.

		% Correctly Classified			
Comparison Groups	VOCs Included in the Analysis	Naïve Bayes	Logistic	RF	AUC for RF
**Breath concentrations (C_exhaled_)**					
**Ca^+^ vs. HC**	All 19 VOCs	72.1	80.7	88.5	0.940
	* Ethylbenzene, Toluene, Styrene, Benzene, Cyclohexane, 1-propanol, 2-propanol	70.2	78.8	83.7	0.908
	** Ethylbenzene, Toluene, styrene, 1-propanol, 2-propanol, hexane	73.1	74.3	76.3	0.839
**Smokers** **Ca^+^ vs. HC**	All 19 VOCs	71.4	71.4	85.7	0.934
	* Ethylbenzene, Toluene, Styrene, Benzene, Cyclohexane, 1-propanol, 2-propanol	85.7	71.4	89.2	0.769
	** Ethylbenzene, Toluene, styrene, 1-propanol, 2-propanol, hexane	85.7	82.1	82.1	0.822
**Non-smokers** **Ca^+^ vs. HC**	All 19 VOCs	73.7	82.9	895	0.970
	* Ethylbenzene, Toluene, Styrene, Benzene, Cyclohexane, 1-propanol, 2-propanol	69.7	80.2	86.8	0.910
	** Ethylbenzene, Toluene, styrene, 1-propanol, 2-propanol, hexane	67.1	76.3	77.6	0.898
**Ca^+^ vs. Ca^−^**	All 19 VOCs	40.4	41.6	35.9	0.342
**Breath Subtracts** **ΔC = C_exhaled_ − C_ambient_**					
**Ca^+^ vs. HC**	All 19 VOCs	55.7	80.8	78.8	0.888
	* Ethylbenzene, Toluene, Styrene,1-propanol, 2-propanol, hexane, ethylbutyrate	67.3	76.9	78.8	0.86
	** Ethylbenzene, Toluene, Styrene, 1-propanol, ethylbutyrate	65.4	78.8	78.8	0.853
**Smokers** **Ca^+^ vs. HC**	All 19 VOCs	71.4	71.4	85.7	0.831
	* Ethylbenzene, Toluene, Styrene,1-propanol, 2-propanol, hexane, ethylbutyrate	85.7	85.7	89.3	0.947
	** Ethylbenzene, Toluene, Styrene,1-propanol, ethylbutyrate	89.3	82.1	85.7	0.95
**Non-smokers** **Ca^+^ vs. HC**	All 19 VOCs	64.5	82.9	78.9	0.892
	* Ethylbenzene, Toluene, Styrene,1-propanol, 2-propanol, hexane, ethylbutyrate	77.6	77.6	81.6	0.901
	** Ethylbenzene, Toluene, Styrene, 1-propanol, ethylbutyrate	76.3	76.3	84.2	0.881
**Ca^+^ vs. Ca^−^**	All 19 VOCs	43.8	52.8	41.6	0.266

* VOCs identified from the Mann–Whitney test. ** Informative VOCs identified by feature selection module within Weka. The last column shows the area under the curve for the random forest method. All methods were implemented in WEKA with default parameters.

## Supplementary Materials

The following are available online at https://www.mdpi.com/2218-1989/10/8/317/s1. Supplementary Table S1: Characteristics of study participants. Supplementary Table S2: Detection frequencies, median (IQR), minimum, and maximum concentrations (ng/L air) in exhaled breath of study’s participants. Supplementary Table S3: Summary of correct classification, based on naïve Bayes, logistic regression, and random forest machine learning methods. Supplementary Table S4: Range of calibration, detection limits (LOD), quantification limits (LOQ), and the linearity of the detector response (R2) and coefficients of variation for 5 consecutive calibration curves. Supplementary Figures S1–S17: Median breath concentrations of lung cancer patients and healthy controls in comparison with the corresponding ambient air concentrations.
