# Supplementary material for: Target Analysis of Volatile Organic Compounds in Exhaled Breath for Lung Cancer Discrimination from Other Pulmonary Diseases and Healthy Persons
Source: Metabolites. 2020 Aug 3;10(8):317. doi: 10.3390/metabo10080317 (PMC7464039; doi:10.3390/metabo10080317)

**Supplementary figures (SF1-SF17)**

**Median breath concentrations of lung cancer patients and healthy controls in comparison with the corresponding ambient air concentrations**

*Data for thiophene and 1-butanol are not presented due to low detection frequency.

Figure SF 1:ISOPRENE


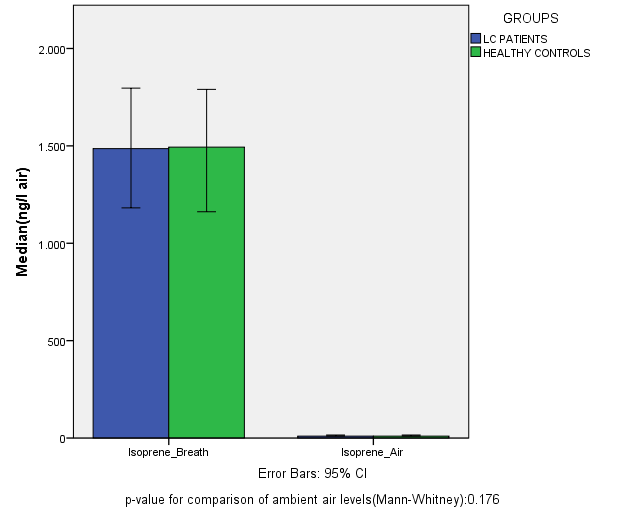


Figure SF 2: ACETONE


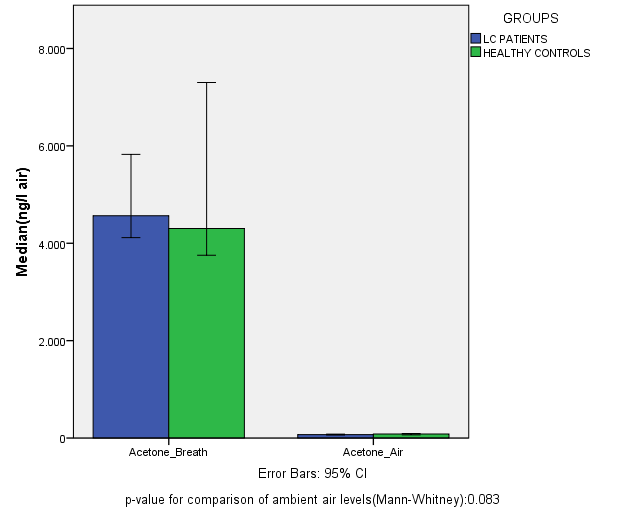


Figure SF 3: 2-PROPANOL

**
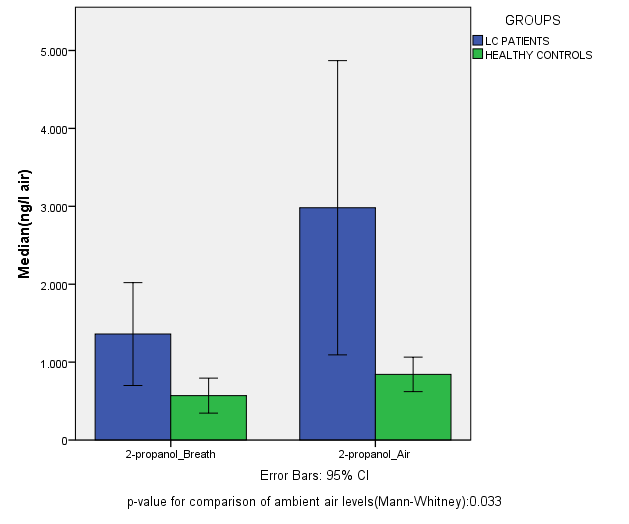
**

Figure SF 4: 1-PROPANOL


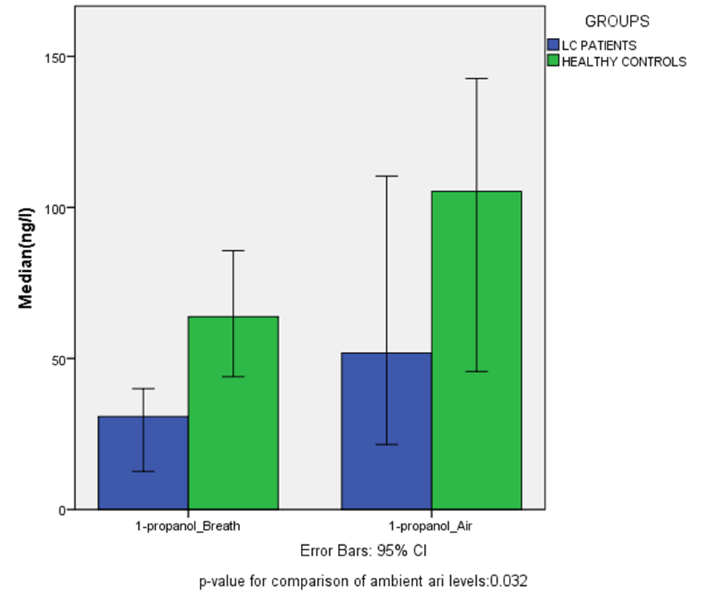


Figure SF 5:HEXANE


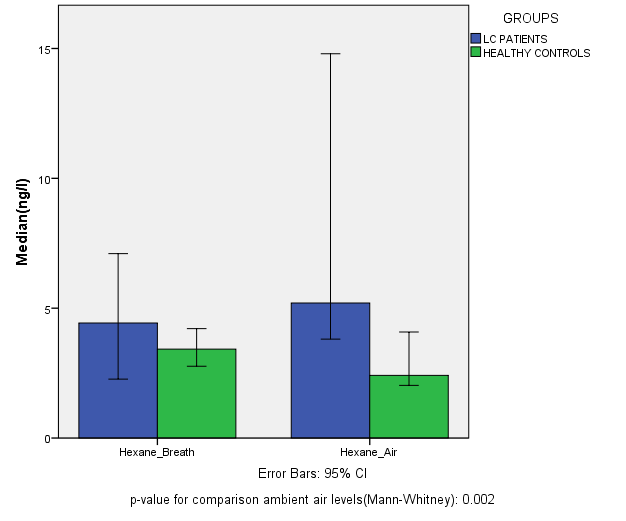


Figure SF 6: 2-BUTANONE


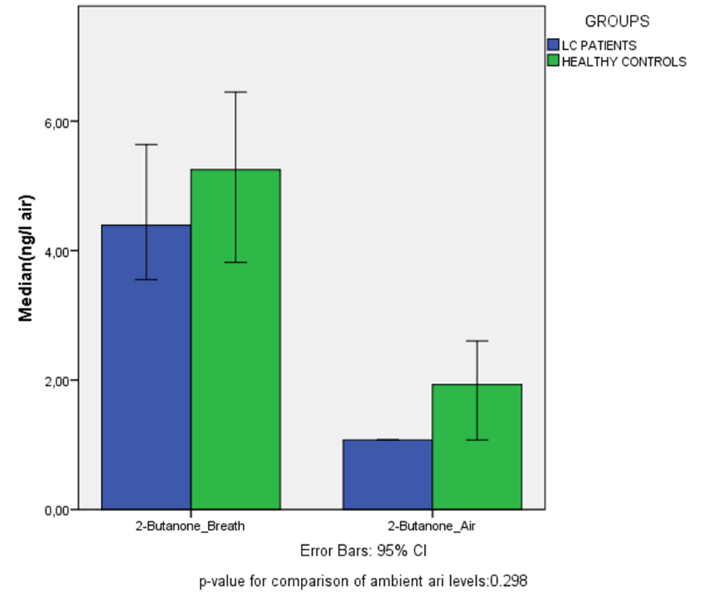


Figure SF 7: CYCLOHEXANE


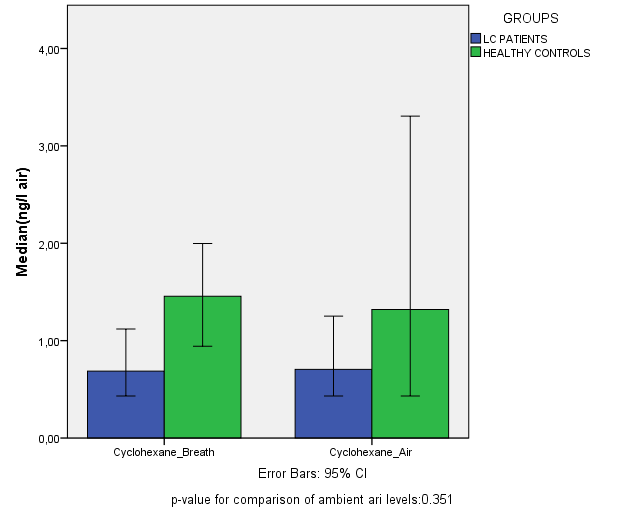


Figure SF 8: BENZENE


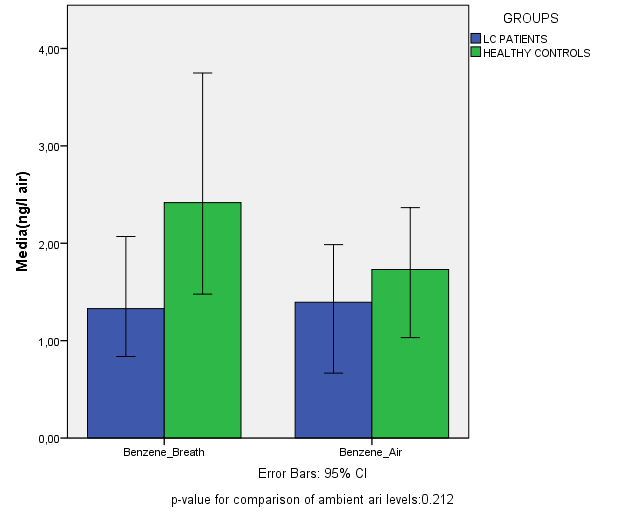


Figure SF 9: TOLUENE


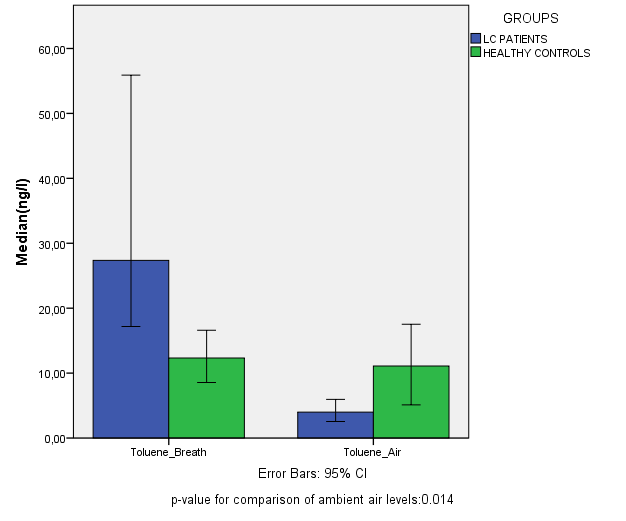


Figure SF 10:OCTANE


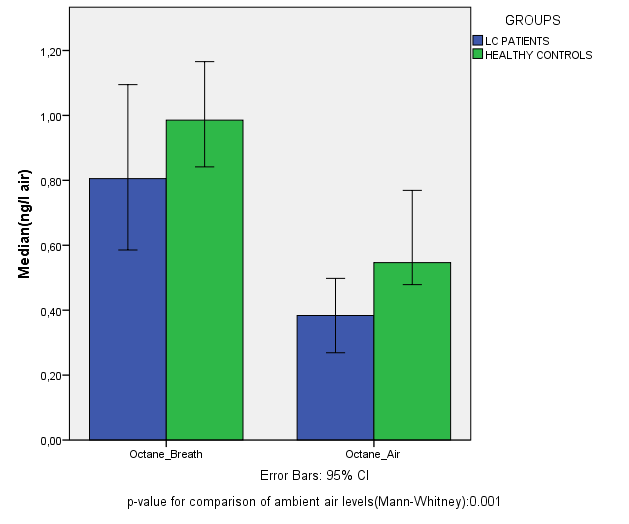


Figure SF 11: ETHYLBUTYRATE


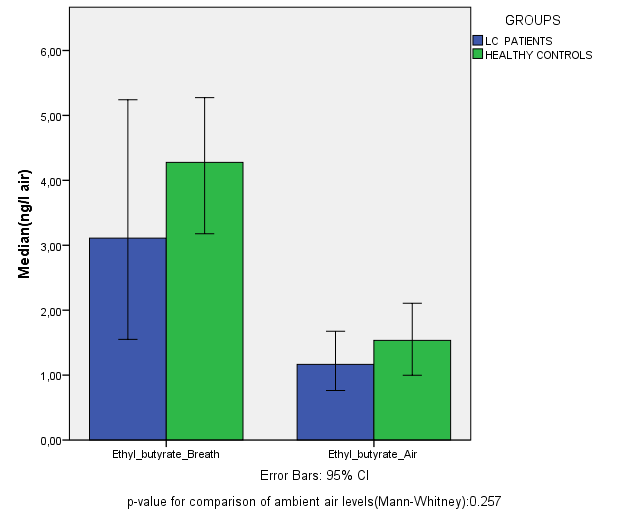


Figure SF 12: HEXANAL


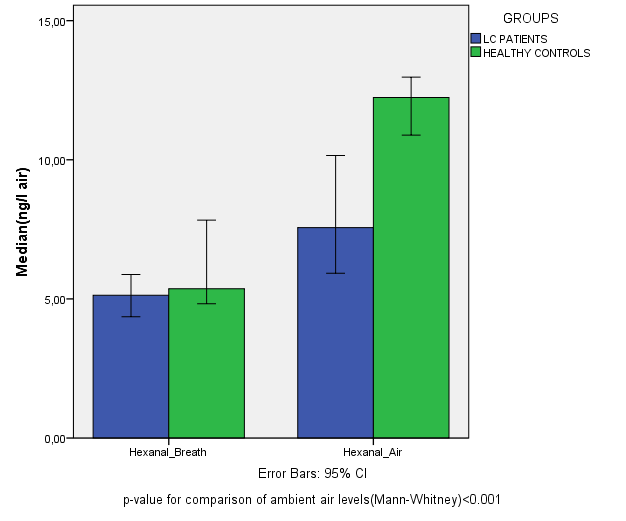


Figure SF 13: ETHYLBENZENE


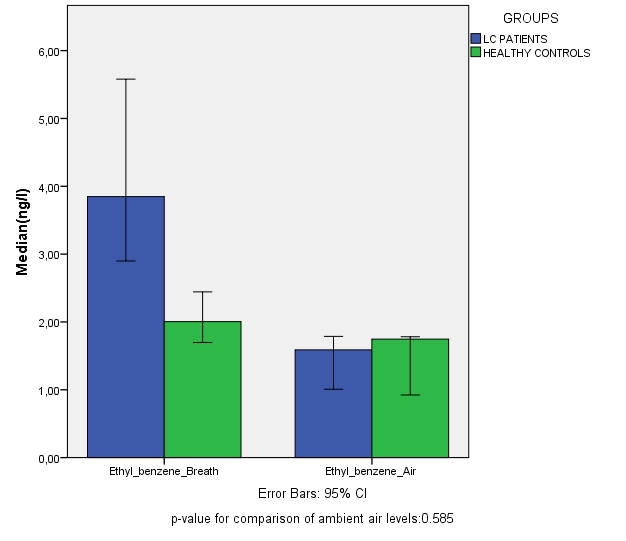


Figure SF 14:STYRENE


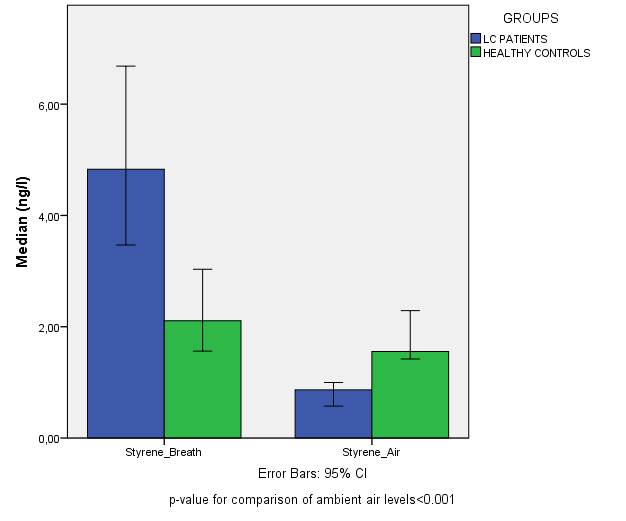


Figure SF 15:CYCLOHEXANONE


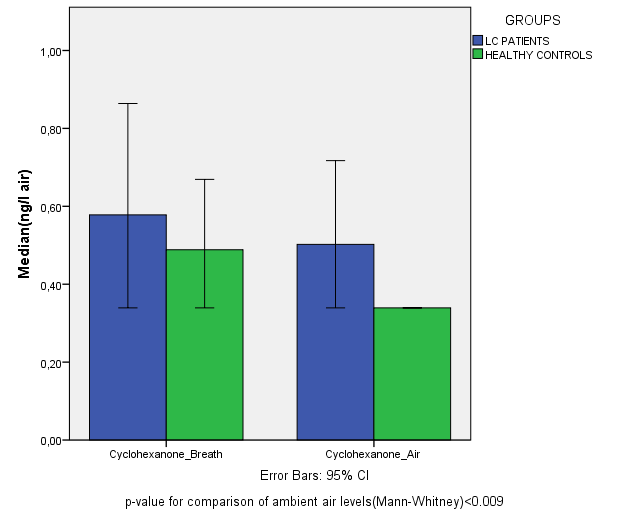


Figure SF 16: OCTANAL


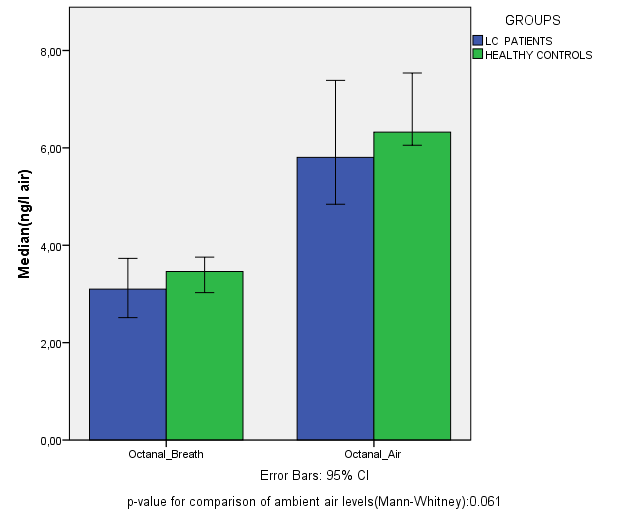


Figure SF 17:NONANAL


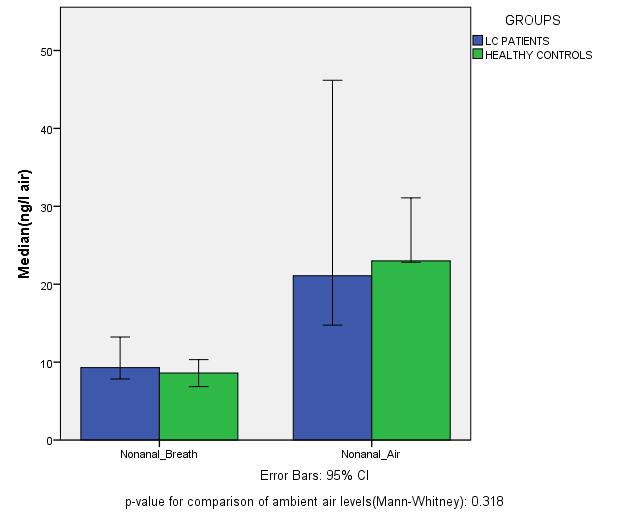

Supplement: Supplementary file 1 [file metabolites-10-00317-s001.zip › SUPPLEMENTARY FIGURES V2.docx]
