# Supplementary material for: Target Analysis of Volatile Organic Compounds in Exhaled Breath for Lung Cancer Discrimination from Other Pulmonary Diseases and Healthy Persons
Source: Metabolites. 2020 Aug 3;10(8):317. doi: 10.3390/metabo10080317 (PMC7464039; doi:10.3390/metabo10080317)
Supplement: Supplementary file 1 [file metabolites-10-00317-s001.zip › Supplementary tablesv1.docx]

| **Supplementary Table 1: Characteristics of study participants** | | | | | | | | |
| --- | --- | --- | --- | --- | --- | --- | --- | --- |
| A/A | group | Gender | Age | BMI | smoking | diagnosis | CT findings | Symptoms |
| 1 | Ca(-) | Female | 70 | 41,21 | Active Smoker | NO CANCER | pleural effusion and pulmonary opacities | chest pain |
| 2 | Ca(-) | Male | 66 | 28,01 | Active Smoker | NO CANCER | pulmonary opacities | coughchest pain |
| 3 | Ca(-) | Male | 62 | 19,03 | Former Smoker | NO CANCER | pulmonary cavities | dyspnea |
| 4 | Ca(-) | Male | 65 | 26,73 | Former Smoker | NO CANCER | normal | bloody sputum |
| 5 | Ca(-) | Male | 50 | 24,57 | Former Smoker | NO CANCER | pulmonary cavities | fever |
| 6 | Ca(-) | Male | 36 | 22,09 | Active Smoker | NO CANCER | pulmonary cavities | cough |
| 7 | Ca(-) | Female | 75 | 26,17 | never | NO CANCER | pulmonary opacities and nodules | cough |
| 8 | Ca(-) | Male | 70 | 27,78 | Former Smoker | NO CANCER | normal | bloody sputum |
| 9 | Ca(-) | Male | 72 | 24,16 | Active Smoker | NO CANCER | pulmonary opacities | cough |
| 10 | Ca(-) | Male | 64 | 23,78 | Former Smoker | NO CANCER | pulmonary mass | coughweight loss |
| 11 | Ca(-) | Male | 70 | 27,55 | Former Smoker | NO CANCER | pulmpnary opacities and lymph node enlargement | dyspnea |
| 12 | Ca(-) | Female | 68 | 28,44 | never | NO CANCER | pulmonary opacities | cough |
| 13 | Ca(-) | Male | 71 | 29,38 | never | NO CANCER | pulmonary opacities | cough |
| 14 | Ca(-) | Male | 37 | 20,76 | Active Smoker | NO CANCER | pulmonary opacities and cavity | cough |
| 15 | Ca(-) | Male | 46 | 27,68 | never | NO CANCER | lymph node enlargement | cough |
| 16 | Ca(-) | Male | 56 | 29,61 | Former Smoker | NO CANCER | pulmonary opacities | bloody sputumfever |
| 17 | Ca(-) | Male | 61 | 33,56 | Former Smoker | NO CANCER | pulmonary opacities | fever |
| 18 | Ca(-) | Male | 68 | 37,18 | Former Smoker | NO CANCER | lymph node enlargement |  |
| 19 | Ca(-) | Male | 39 | 31,56 | Former Smoker | NO CANCER | lymph node enlargement |  |
| 20 | Ca(-) | Female | 56 | 32,05 | never | NO CANCER | lymph node enlargement (sarcoidosis) |  |
| 21 | Ca(-) | Female | 73 | 31,14 | never | NO CANCER | pulmonary opacities and nodules ( frequent respiratoy infections) | cough |
| 22 | Ca(-) | Male | 72 | 30,42 | Active Smoker | NO CANCER | pulmonary opacities | cough |
| 23 | Ca(-) | Female | 81 | 30,22 | Active Smoker(ocassionally) | NO CANCER | pulmonary opacities |  |
| 24 | Ca(-) | Male | 84 | 17,72 | Active Smoker | NO CANCER | pulmonary opacities and nodules | cough |
| 25 | Ca(-) | Male | 64 | 26,83 | Former Smoker | NO CANCER | pulmonary opacities |  |
| 26 | Ca(-) | Male | 73 | 27,77 | Former Smoker | NO CANCER | pulmonary nodules (random finding) |  |
| 27 | Ca(-) | Male | 69 | 19,38 | Former Smoker | NO CANCER | pulmonary opacities | bloody sputum |
| 28 | Ca(-) | Male | 65 | 26,42 | Former Smoker | NO CANCER | pulmonary nodule |  |
| 29 | Ca(-) | Male | 43 | 23,84 | Active Smoker(ocassionally) | NO CANCER | pulmonary opacities |  |
| 30 | Ca(-) | Male | 71 | 30,93 | Former Smoker | NO CANCER | pulmonary opacities | cough |
| 31 | Ca(-) | Male | 79 | 25,61 | Active Smoker | NO CANCER | lymph node enlargement |  |
| 32 | Ca(-) | Male | 80 | 26,89 | Former Smoker | NO CANCER | pulmonary nodules | cough |
| 33 | Ca(-) | Male | 75 | 25,59 | Former Smoker | NO CANCER | pulmonary opacities | cough |
| 34 | Ca(-) | Male | 73 | 23,18 | Former Smoker | NO CANCER | lymph node enlargement and pulmonary opacities | cough |
| 35 | Ca(-) | Male | 72 | 31,25 | Former Smoker | NO CANCER | pulmonary nodules | chest pain |
| 36 | Ca(-) | Male | 78 | 30,48 | Former Smoker | NO CANCER | pulmonary opacities | dyspnea |
| 37 | Ca(-) | Male | 67 | 25,56 | Former Smoker | NO CANCER | lymph node enlargement and pulmonary nodules | cough |
| 38 | Ca(-) | Female | 57 | 26,45 | never | NO CANCER | normal (for sarcoidosis) | cough |
| 39 | Ca(+) | Male | 79 | 31,14 | Former Smoker | NSCL | pulmonary opacities | bloody sputum |
| 40 | Ca(+) | Male | 64 | 26,12 | Former Smoker | NSCL | pulmonary opacities | hemoptysis |
| 41 | Ca(+) | Male | 77 | 28,69 | Active Smoker | NSCL | pulmonary opacities | bloody sputum |
| 42 | Ca(+) | Female | 64 | 33,20 | never | NSCL | pulmonary opacities |  |
| 43 | Ca(+) | Female | 64 | 19,53 | Former Smoker | NEYROEND NEOPLASMA | pulmonary nodules | dyspneacough |
| 44 | Ca(+) | Male | 77 | 23,46 | Former Smoker | NSCL | pulmonary opacities | dyspneacough |
| 45 | Ca(+) | Male | 80 | 29,94 | Former Smoker | NSCL | pulmonary opacities | dyspneacough |
| 46 | Ca(+) | Male | 67 | 30,86 | Former Smoker | NSCL | pulmonary opacities | wheezing |
| 47 | Ca(+) | Male | 78 | 23,74 | Former Smoker | NSCL | pulmonary mass |  |
| 48 | Ca(+) | Male | 63 | 29,05 | Former Smoker | NSCL | pulmonary opacities |  |
| 49 | Ca(+) | Female | 57 | 21,72 | never | NSCL | pulmonary opacities | cough |
| 50 | Ca(+) | Male | 74 | 36,59 | Former Smoker | NSCL | pulmonary opacities | cough |
| 51 | Ca(+) | Female | 69 | 25,86 | Active Smoker | NSCL | lymph node enlargement | cough |
| 52 | Ca(+) | Male | 71 | 27,68 | Former Smoker | SCLC | lymph node enlargement and pulmonary opacities |  |
| 53 | Ca(+) | Male | 81 | 30,39 | Former Smoker | NSCL | pulmonary opacities | bloody sputum |
| 54 | Ca(+) | Male | 66 | 19,03 | Former Smoker | NSCL | pulmonary cavities | coughchest pain |
| 55 | Ca(+) | Male | 68 | 23,66 | Active Smoker | SCLC | pulmonary mass | weight loss |
| 56 | Ca(+) | Male | 73 | 30,47 | Former Smoker | NSCL | pulmonary opacities | cough |
| 57 | Ca(+) | Male | 68 | 24,44 | Active Smoker | NSCL | pulmonary opacities and lymph node enlargement |  |
| 58 | Ca(+) | Male | 86 | 20,81 | Former Smoker | NSCL | pulmonary opacities | dyspnea |
| 59 | Ca(+) | Male | 64 | 19,10 | Former Smoker | NSCL | pulmonary opacities |  |
| 60 | Ca(+) | Male | 59 | 29,74 | Former Smoker | NSCL | pulmonary opacities | hemoptysis |
| 61 | Ca(+) | Male | 82 | 24,39 | Active Smoker | NSCL | pulmonary cavities | bloody sputumfever |
| 62 | Ca(+) | Male | 72 | 27,55 | Former Smoker | SCLC | pulmonary opacities | cough |
| 63 | Ca(+) | Male | 78 | 24,07 | Former Smoker | SCLC | pulmonary opacities | coughchest pain |
| 64 | Ca(+) | Female | 66 | 25,71 | Active Smoker(ocassionally) | NSCL | lymph node enlargement and pulmonary mass |  |
| 65 | Ca(+) | Male | 67 | 24,24 | Former Smoker | SCLC | pulmonary opacities |  |
| 66 | Ca(+) | Male | 69 | 24,22 | Former Smoker | NSCL | pulmonary opacities | dyspnea |
| 67 | Ca(+) | Male | 73 | 21,55 | Former Smoker | SCLC | pulmonary opacities | dyspneacoughchest pain |
| 68 | Ca(+) | Female | 63 | 23,26 | Former Smoker | NSCL | pulmonary opacities | cough |
| 69 | Ca(+) | Male | 83 | 28,73 | Former Smoker | NSCL | pulmonary opacities | cough |
| 70 | Ca(+) | Male | 84 | 26,64 | Former Smoker | NSCL | pulmonary opacities (random finding) |  |
| 71 | Ca(+) | Male | 60 | 32,85 | never | NSCL | pulmonary opacities (random finding) |  |
| 72 | Ca(+) | Male | 77 | 22,34 | Former Smoker | NSCL | pulmonary opacities | coughbloody sputum |
| 73 | Ca(+) | Male | 69 | 29,74 | Active Smoker | NSCL | pulmonary opacities | cough |
| 74 | Ca(+) | Male | 71 | 24,22 | Former Smoker | NSCL | pulmonary mass |  |
| 75 | Ca(+) | Male | 63 | 20,57 | Former Smoker | NSCL | pulmonary opacities | cough |
| 76 | Ca(+) | Male | 71 | 30,78 | Former Smoker | NSCL | lymph node enlargement |  |
| 77 | Ca(+) | Male | 75 | 26,26 | Former Smoker | NSCL | pulmonary opacities | cough |
| 78 | Ca(+) | Male | 80 | 29,02 | Former Smoker | NSCL | pulmonary opacities | bloody sputum |
| 79 | Ca(+) | Female | 46 | 23,14 | Former Smoker | NSCL | pulmonary opacities | cough |
| 80 | Ca(+) | Male | 56 | 33,31 | Former Smoker | SCLC | pulmonary opacities | bloody sputum |
| 81 | Ca(+) | Male | 80 | 29,39 | Former Smoker | NSCL | pulmonary opacities | chest pain |
| 82 | Ca(+) | Male | 79 | 19,41 | Former Smoker | NSCL | pulmonary opacities |  |
| 83 | Ca(+) | Male | 68 | 35,49 | Former Smoker | NSCL | pulmonary opacities | bloody sputum |
| 84 | Ca(+) | Male | 74 | 31,22 | Former Smoker | NSCL | pulmonary opacities and lymph node enlargement | cough |
| 85 | Ca(+) | Male | 73 | 23,15 | Former Smoker | SCLC | pulmonary opacities |  |
| 86 | Ca(+) | Male | 76 | 23,46 | Former Smoker | NSCL | pulmonary opacities | dyspnea |
| 87 | Ca(+) | Male | 75 | 25,95 | Former Smoker | NSCL | pulmonary opacities |  |
| 88 | Ca(+) | Male | 68 | 30,49 | Active Smoker | NSCL | lymph node enlargement and pulmonary mass |  |
| 89 | Ca(+) | Male | 69 | 23,78 | Former Smoker | NSCL | pulmonary opacities | bloody sputum |
| 90 | HC | Female | 48 | 30,86 | Active smoker |  |  |  |
| 91 | HC | Male | 66 | 23,32 | Former smoker |  |  |  |
| 92 | HC | Male | 58 | 29,56 | Former smoker |  |  |  |
| 93 | HC | Male | 64 | 31,83 | Active smoker |  |  |  |
| 94 | HC | Female | 57 | 44,14 | Former smoker |  |  |  |
| 95 | HC | Male | 82 | 26,99 | Active smoker |  |  |  |
| 96 | HC | Female | 66 | 29,38 | Former smoker |  |  |  |
| 97 | HC | Male | 71 | 26,95 | Former smoker |  |  |  |
| 98 | HC | Male | 56 | 44,08 | Former smoker |  |  |  |
| 99 | HC | Female | 72 | 26,06 | Former smoker |  |  |  |
| 100 | HC | Female | 54 | 32,69 | never |  |  |  |
| 101 | HC | Male | 65 | 20,76 | Active smoker |  |  |  |
| 102 | HC | Male | 60 | 24,77 | Active smoker |  |  |  |
| 103 | HC | Male | 63 | 25,61 | Active smoker |  |  |  |
| 104 | HC | Male | 58 | 31,46 | Active smoker |  |  |  |
| 105 | HC | Male | 53 | 23,51 | Active smoker |  |  |  |
| 106 | HC | Female | 51 | 21,26 | Active smoker |  |  |  |
| 107 | HC | Male | 41 | 31,38 | never |  |  |  |
| 108 | HC | Female | 48 | 18,26 | never |  |  |  |
| 109 | HC | Male | 68 | 25,31 | Former smoker |  |  |  |
| 110 | HC | Female | 64 | 28,69 | Active smoker |  |  |  |
| 111 | HC | Female | 66 | 27,55 | Former smoker |  |  |  |
| 112 | HC | Male | 59 | 27,04 | Active smoker |  |  |  |
| 113 | HC | Male | 72 | 30,42 | Former smoker |  |  |  |
| 114 | HC | Male | 85 | 26,64 | Former smoker |  |  |  |
| 115 | HC | Female | 43 | 23,05 | never |  |  |  |
| 116 | HC | Male | 78 | 31,14 | Former smoker |  |  |  |
| 117 | HC | Male | 64 | 16,33 | Active smoker |  |  |  |
| 118 | HC | Male | 88 | 26,73 | Active smoker |  |  |  |
| 119 | HC | Female | 62 | 37,10 | Active smoker |  |  |  |
| 120 | HC | Female | 75 | 25,39 | never |  |  |  |
| 121 | HC | Female | 70 | 33,66 | never |  |  |  |
| 122 | HC | Female | 71 | 26,04 | never |  |  |  |
| 123 | HC | Male | 72 | 20,42 | Active smoker |  |  |  |
| 124 | HC | Male | 70 | 22,86 | never |  |  |  |
| 125 | HC | Male | 67 | 33,80 | never |  |  |  |
| 126 | HC | Male | 73 | 29,05 | Former smoker |  |  |  |
| 127 | HC | Female | 61 | 30,76 | Active smoker |  |  |  |
| 128 | HC | Male | 79 | 29,38 | Former smoker |  |  |  |
| 129 | HC | Male | 65 | 26,78 | Active smoker |  |  |  |
| 130 | HC | Male | 67 | 26,83 | Former smoker |  |  |  |
| 131 | HC | Male | 81 | 25,01 | Former smoker |  |  |  |
| 132 | HC | Male | 79 | 29,41 | never |  |  |  |
| 133 | HC | Female | 68 | 20,22 | Former smoker |  |  |  |
| 134 | HC | Female | 66 | 28,37 | never |  |  |  |
| 135 | HC | Male | 79 | 25,35 | Former smoker |  |  |  |
| 136 | HC | Male | 77 | 27,73 | never |  |  |  |
| 137 | HC | Male | 66 | 35,38 | never |  |  |  |
| 138 | HC | Male | 68 | 23,01 | Active smoker |  |  |  |
| 139 | HC | Male | 74 | 36,51 | Active smoker |  |  |  |
| 140 | HC | Male | 82 | 25,50 | Active smoker |  |  |  |
| 141 | HC | Male | 86 | 27,73 | never |  |  |  |
| 142 | HC | Male | 63 | 30,37 | never |  |  |  |

| **Supplementary table 3: Detection frequencies, median (IQR), minimum, maximum concentrations (ng/l air) in exhaled breath of study's participants** | | | | | | |
| --- | --- | --- | --- | --- | --- | --- |
|  | **% detected** | **Median** | **Minimum** | **Percentile 25** | **Percentile 75** | **Maximum** |
| **isoprene** | **100** | **1524** | **356** | **1042** | **2017** | **5552** |
| **acetone** | **100** | **4441** | **935** | **2843** | **7916** | **96279** |
| **isopropanol** | **100** | **437** | **85** | **276** | **678** | **10293** |
| **hexane** | **82,40** | **4,24** | **nd** | **2,17** | **11,80** | **342,31** |
| **1-propanol** | **99,30** | **39,49** | **nd** | **12,78** | **82,45** | **1844,89** |
| **2-butanone** | **98,60** | **5,10** | **nd** | **3,27** | **7,00** | **941,17** |
| **cyclohexane** | **69,90** | **0,96** | **nd** | **nd** | **2,14** | **16,86** |
| **benzene** | **92,30** | **1,73** | **nd** | **0,84** | **3,95** | **76,33** |
| **thiophene** | **1,40** | **nd** | **nd** | **nd** | **nd** | **0,95** |
| **1-butanol** | **40,10** | **nd** | **nd** | **nd** | **1,15** | **4,51** |
| **toluene** | **97,90** | **19,04** | **nd** | **10,25** | **42,69** | **148,73** |
| **octane** | **90,80** | **0,94** | **nd** | **0,58** | **1,38** | **10,57** |
| **ethyl butyrate** | **87,30** | **3,58** | **nd** | **1,22** | **5,97** | **46,66** |
| **hexanal** | **97,90** | **5,23** | **nd** | **3,95** | **7,68** | **54,96** |
| **ethylbenzene** | **93,00** | **2,78** | **nd** | **1,68** | **4,63** | **15,24** |
| **styrene** | **99,30** | **3,47** | **nd** | **1,48** | **6,68** | **19,73** |
| **cyclohexanone** | **54,20** | **0,51** | **nd** | **nd** | **1,06** | **7,46** |
| **n-octanal** | **97,90** | **3,25** | **nd** | **2,21** | **4,42** | **33,44** |
| **nonanal** | **100,00** | **8,40** | **2,06** | **5,83** | **13,86** | **328,44** |

| **Supplementary table 3: Summary of correct classification, based on Naïve Bayes, Logistic Regression and Random Forest Machine learning methods.** | | | | | | | | |
| --- | --- | --- | --- | --- | --- | --- | --- | --- |
|  |  |  |  |  | **%Correctly classified** | | |  |
| **Comparison groups** | **variable examined** | **No of VOCS** | **Group1** | **Group2** | **Naïve_Bayes** | **Logistic** | **RF** | **AUC for RF** |
| **Ca(+) vs HC** | **Breath** | **19** | **51** | **53** | **72,1** | **80,7** | **88,5** | **0,94** |
| **Ca(+) vs HC [smokers]** | **Breath** | **19** | **8** | **20** | **71,4** | **71,4** | **85,7** | **0,934** |
| **Ca(+) vs HC [nonsmokers]** | **Breath** | **19** | **43** | **33** | **73,7** | **82,9** | **89,5** | **0,97** |
| **Ca(+) vs Ca(-)** | **Breath** | **19** | **51** | **38** | **40,4** | **41,6** | **35,9** | **0,342** |
| **Ca(+) vs Ca(-) [smokers]** | **Breath** | **19** | **8** | **10** | **38,9** | **16,7** | **33,3** | **0,181** |
| **Ca(+) vs Ca(-) [nonsmokers]** | **Breath** | **19** | **43** | **28** | **38** | **59,2** | **45** | **0,459** |
| **Ca(+) vs HC** | **Breath** | **7** | **51** | **53** | **70,2** | **78,8** | **83,7** | **0,908** |
| **Ca(+) vs HC [smokers]** | **Breath** | **7** | **8** | **20** | **85,7** | **71,4** | **89,2** | **0,769** |
| **Ca(+) vs HC [nonsmokers]** | **Breath** | **7** | **43** | **33** | **69,7** | **80,2** | **86,8** | **0,912** |
| **Ca(+) vs HC** | **Breath** | **6** | **51** | **53** | **73,1** | **74** | **76** | **0,839** |
| **Ca(+) vs HC [smokers]** | **Breath** | **6** | **8** | **20** | **85,7** | **82,1** | **82,1** | **0,822** |
| **Ca(+) vs HC [nonsmokers]** | **Breath** | **6** | **43** | **33** | **67,1** | **76,3** | **77,6** | **0,898** |
| **Ca(-) vs HC** | **Breath** | **19** | **38** | **53** | **68,1** | **76,9** | **82,4** | **0,906** |
| **All patients vs HC** | **Breath** | **19** | **89** | **53** | **72,5** | **83,1** | **82,4** | **0,948** |
| **Ca(+) vs HC** | **Gradient** | **19** | **51** | **53** | **55,7** | **80,8** | **78,8** | **0,888** |
| **Ca(+) vs HC [smokers]** | **Gradient** | **19** | **8** | **20** | **71,4** | **71,4** | **85,7** | **0,831** |
| **Ca(+) vs HC [nonsmokers]** | **Gradient** | **19** | **43** | **33** | **64,5** | **82,9** | **78,9** | **0,892** |
| **Ca(+) vs Ca(-)** | **Gradient** | **19** | **51** | **38** | **43,8** | **52,8** | **41,6** | **0,266** |
| **Ca(+) vs Ca(-) [smokers]** | **Gradient** | **19** | **8** | **10** | **55,5** | **66,6** | **44,4** | **0,387** |
| **Ca(+) vs Ca(-) [nonsmokers]** | **Gradient** | **19** | **43** | **28** | **33,8** | **49,3** | **39,4** | **0,274** |
| **Ca(+) vs HC** | **Gradient** | **7** | **51** | **53** | **67,3** | **76,9** | **78,8** | **0,86** |
| **Ca(+) vs HC [smokers]** | **Gradient** | **7** | **8** | **20** | **85,7** | **85,7** | **89,3** | **0,947** |
| **Ca(+) vs HC [nonsmokers]** | **Gradient** | **7** | **43** | **33** | **77,6** | **77,6** | **81,6** | **0,901** |
| **Ca(+) vs HC** | **Gradient** | **5** | **51** | **53** | **65,4** | **78,8** | **78,8** | **0,853** |
| **Ca(+) vs HC [smokers]** | **Gradient** | **5** | **8** | **20** | **89,3** | **82,1** | **85,7** | **0,95** |
| **Ca(+) vs HC[nonsmokers[** | **Gradient** | **5** | **43** | **33** | **76,3** | **76,3** | **84,2** | **0,881** |
| **Ca(-) vs HC** | **Gradient** | **19** | **51** | **38** | **59,3** | **74,7** | **80,2** | **0,866** |
| **All patients vs HC** | **Gradient** | **19** | **89** | **53** | **66,9** | **82,4** | **81** | **0,893** |
| Ca+: Patients diagnosed with Lung Cancer, Ca-: Patients with pathological CT findings not diagnosed with Lung cancer by histological/cytological examination, HC : Healthy Controls. The last column shows the Area Under the Curve for the Random Forest method. All methods were implemented in WEKA with default parameters. | | | | | | | | |

| **Supplementary table 4: Range of calibration, detection limits (LOD), quantification limits (LOQ) and the linearity of the detector response (R2) and coefficients of variation for 5 consecutive calibration curves** | | | | | | | |
| --- | --- | --- | --- | --- | --- | --- | --- |
| **Substance** | **Calibration range(ng/l air)** | **mean R^2^** | **CV%-R^2^** | **mean a(slope)** | **CV%-a** | **LOD(ng/l)** | **LOQ(ng/l)** |
| **isoprene** | **68,1-10896** | **0,9989** | **0,09%** | **4340** | **13,84%** | **56,56** | **186,68** |
| **acetone** | **78,4-12544** | **0,9981** | **0,22%** | **1962** | **11,50%** | **49,44** | **163,16** |
| **isopropanol** | **15,72-1257** | **0,9968** | **0,45%** | **1082** | **14,57%** | **5,83** | **19,23** |
| **hexane** | **0,21-32,8** | **0,9982** | **0,30%** | **921** | **19,69%** | **0,49** | **1,6** |
| **1-propanol** | **0,27-42,7** | **0,9987** | **0,22%** | **5725** | **8,24%** | **0,19** | **0,62** |
| **2-butanone** | **0,26-41,6** | **0,9879** | **1,15%** | **1220** | **22,34%** | **0,46** | **1,52** |
| **cyclohexane** | **0,26-41,6** | **0,9975** | **0,19%** | **2395** | **6,46%** | **0,19** | **0,61** |
| **benzene** | **0,24-46,4** | **0,9981** | **0,16%** | **38861** | **14,90%** | **0,11** | **0,38** |
| **thiophene** | **0,35-56,0** | **0,9987** | **0,11%** | **2128** | **10,10%** | **0,41** | **1,36** |
| **1-butanol** | **0,27-43,2** | **0,9984** | **0,09%** | **9013** | **9,75%** | **0,56** | **1,85** |
| **toluene** | **0,28-44,8** | **0,9972** | **0,34%** | **71838** | **5,92%** | **0,66** | **2,16** |
| **octane** | **0,23-36,8** | **0,9966** | **0,28%** | **6182** | **7,01%** | **0,38** | **1,26** |
| **ethyl butyrate** | **0,29-46,4** | **0,991** | **1,18%** | **3507** | **7,51%** | **0,45** | **1,47** |
| **hexanal** | **0,27-43,2** | **0,992** | **0,52%** | **6156** | **15,74%** | **0,44** | **1,47** |
| **ethylbenzene** | **0,29-45,8** | **0,9951** | **0,50%** | **72527** | **4,78%** | **0,53** | **1,75** |
| **styrene** | **0,30-48,0** | **0,9963** | **0,35%** | **29899** | **5,12%** | **0,53** | **1,75** |
| **cyclohexanone** | **0,31-49,6** | **0,9954** | **0,57%** | **7260** | **16,94%** | **0,48** | **1,58** |
| **n-octanal** | **0,27-43,7** | **0,9924** | **1,04%** | **5001** | **12,27%** | **0,38** | **1,25** |
| **nonanal** | **0,27-43,5** | **0,9911** | **0,61%** | **2155** | **12,09%** | **1,25** | **4,13** |
